# Factors Predicting the Presence of Maternal Cells in Cord Blood and Associated Changes in Immune Cell Composition

**DOI:** 10.3389/fimmu.2021.651399

**Published:** 2021-04-22

**Authors:** Marina El Haddad, Karlin R. Karlmark, Xavier-Côme Donato, Gabriel V. Martin, Florence Bretelle, Nathalie Lesavre, Jean-François Cocallemen, Marielle Martin, Christophe Picard, Jean Roudier, Raoul Desbriere, Nathalie C. Lambert

**Affiliations:** ^1^ INSERM UMRs 1097 Arthrites Autoimmunes, Aix Marseille Université, Marseille, France; ^2^ Department of Obstetrics and Gynecology, St Joseph Hospital, Marseille, France; ^3^ Department of Gynaecology and Obstetrics, Pôle Femme Enfant, AP-HM, Assistance Publique-Hôpitaux de Marseille, AMU, Aix-Marseille Université, Marseille, France; ^4^ CIC1409, AMU, AP-HM, Marseille, France; ^5^ Centre National de la Recherche Scientifique (CNRS) UMR7268 (ADES), “Biologie des Groupes Sanguins”, Marseille, France; ^6^ Etablissement Français du Sang PACA Corse, Immunogenetics Laboratory, Marseille, France; ^7^ Service de Rhumatologie, Hôpital Sainte Marguerite, AP-HM, Marseille, France

**Keywords:** cord blood, maternal microchimerism, PAPP-A, HLA compatibility, NK cells, transplantation

## Abstract

**Background:**

Cord blood (CB) samples are increasingly used as a source of hematopoietic stem cells in transplantation settings. Maternal cells have been detected in CB samples and their presence is associated with a better graft outcome. However, we still do not know what influences the presence of maternal microchimerism (MMc) in CB samples and whether their presence influences CB hematopoietic cell composition.

**Patients and Methods:**

Here we test whether genetic, biological, anthropometric and/or obstetrical parameters influence the frequency and/or quantity of maternal Mc in CB samples from 55 healthy primigravid women. Mc was evaluated by targeting non-shared, non-inherited Human Leukocyte Antigen (HLA)-specific real-time quantitative PCR in whole blood and four cell subsets (T, B lymphocytes, granulocytes and/or hematopoietic progenitor cells). Furthermore CB samples were analyzed for their cell composition by flow cytometry and categorized according to their microchimeric status.

**Results:**

MMc was present in 55% of CB samples in at least one cell subset or whole blood, with levels reaching up to 0.3% of hematopoietic progenitor cells. Two factors were predictive of the presence of MMc in CB samples: high concentrations of maternal serological Pregnancy-Associated-Protein-A at first trimester of pregnancy (*p=0.018*) and feto-maternal HLA-A and/or –DR compatibility (*p=0.009* and *p=0.01* respectively). Finally, CB samples positive for MMc were significantly enriched in CD56+ cells compared to CB negative for MMc.

**Conclusions:**

We have identified two factors, measurable at early pregnancy, predicting the presence of maternal cells in CB samples at delivery. We have shown that MMc in CB samples could have an influence on the hematopoietic composition of fetal cells. CD56 is the phenotypic marker of natural killer cells (NK) and NK cells are known to be the main effector for graft versus leukemia reactions early after hematopoietic stem cell transplantation. These results emphasize the importance of MMc investigation for CB banking strategies.

## Introduction

Umbilical Cord Blood (CB), obtained at the time of delivery, is a good source of hematopoietic stem cells and a useful tool for stem cell transplantation for a variety of hematological and non-hematological malignancies and disorders (1, 2). Compared to the conventional adult stem cell sources of bone marrow and peripheral blood, CB are associated with lower incidences of acute and chronic graft versus host disease (GVHD), while maintaining good graft versus leukemia (GVL) activity (3, 4), possibly because of their tolerogenic cell composition, with a majority of naïve T cells and a high proportion of highly suppressive regulatory T cells (5, 6).

Moreover, contrary to what was believed and taught for a long time, during pregnancy, feto-maternal exchange is not only an exchange of nutrients, hormones, antibodies, oxygen and carbon dioxide; a feto-maternal exchange of cells is also established (7). Fetal cells reach the maternal blood stream, leading to a small quantity of persisting cells in the mother called *fetal microchimerism* (Mc) (8). Inversely, maternal cells reach the fetal blood stream to persist as *maternal Mc* in the child (9) and in most cord blood samples (10).

Maternal cells were initially quantified in CB samples mainly because of the fear that they might contribute to the development of GVHD (11). The frequency of maternal nucleated cells in cord blood has been evaluated with variable results ranging from 0% to 100% depending on the sensitivity of detection methods (10, 12–14). The current consensus is that maternal cells are commonly detected in CB samples and amounts are significant (12). Moreover, maternal cells of the CB graft have been recently detected in 19% of 27 unrelated recipients post-CB transplantation (15). Maternal cells may be beneficial as recipients positive for MMc-CB tended to have lower relapse, mortality, and treatment failure than patients negative (15).

During pregnancy, maternal cells are sensitized to the child’s paternally –inherited antigens (IPAs) and can develop a B and T cell immunity against the IPAs of the fetus. Thus, maternal Mc present in CB samples is likely to contribute to superior GVL effects and low rates of disease recurrence when the CB used for hematopoietic stem cell transplantation is matched for IPAs with the unrelated recipient (16).

Conversely, the fetal immune system develops a tolerogenic response toward maternal cells, a tolerance to non-inherited maternal antigens (NIMAs). The NIMAs tolerance has been hypothesized as having a beneficial impact on graft outcome when the recipient shares a mismatch antigen with the CB donor’s mother and this has been supported by two studies showing better transplant outcome after NIMA-matched transplants (17, 18).

As the beneficial role of maternal cells in the fate of the CB transplant is increasingly evidenced (19), here, we propose to identify genetic, biological, anthropometric and obstetrical factors predicting their frequency and quantity. Furthermore we evaluate whether the presence of maternal cells influences the hematopoietic CB cell composition.

## Patients and Methods

### Cord Blood Collection and Maternal Blood Tests

CB samples were collected from 55 healthy primigravid women who had no history of blood transfusion. Samples were obtained by double clamping the umbilical cord segment and drawing CB (~15mL) by venipuncture into lithium heparin tubes from three maternities in Marseille, France (32 from *St Joseph*, 22 from *Nord* and one from *La Conception* maternity). All CB samples were processed within 24 hours from delivery.

All pregnancies were healthy singleton pregnancies with 21 live girls and 34 live boys. Obstetrical, anthropometric and clinical characteristics of mothers and children from whom CB samples were collected are detailed in **Supplementary Table S1**.

A first trimester serum screen (12 ± 2 weeks of amenorrhea), which measures fetal or placental specific proteins, Pregnancy Associated Plasma Protein A (PAPP-A) and free beta human chorionic gonadotropin (β-hCG), has been conducted for 46 of the 55 primigravid women. The level of each serum marker was measured and expressed as the multiple of the median (MoM) of the expected normal median for women with pregnancies of the same gestational day using values established in a previous study (20). Combined with the mother’s age and ultrasound examination to measure nuchal translucency, PAPP-A and β-hCG concentrations are routinely used to assess the risk of Down syndrome or other fetal aneuploidies.

### Collection of Samples and HLA Genotyping

An aliquot of 350µL of blood collected from each primigravid woman at first trimester of pregnancy and of CB collected at delivery, were kept frozen at -40°C. Genomic DNA was extracted from both samples with the EZ1 DNA blood kit (Qiagen, Hilden, Germany) using a Biorobot EZ1 system according to the manufacturer’s instructions.

HLA-A, B and DRB1 genotyping was performed on all DNA samples at Etablissement Français du Sang, Marseilles France, to further investigate maternal Mc in CB samples by HLA-specific PCR.

### Study Approval

The study has received the approval of the ethics committee (CPP Sud-Méditerranée II) and is registered at the INSERM (Biomedical Research Protocol RBM-04-10) and as a collection (DC-2008-327). All participants signed an informed consent form according to the Declaration of Helsinki (21).

### Cord Blood Cell Separation for Chimerism Analyses

CB samples collected in heparin lithium vacutainers were processed to isolate peripheral blood mononuclear cells (PBMC) by gradient centrifugation with Histopaque 1077 (Sigma-Aldrich, MO, USA) and cell subsets were obtained by immuno-magnetic cell sorting (RoboSep™-S, STEMCELL™ Technologies, Canada). B cells, T cells, granulocytes and hematopoietic progenitor cells (HPC) were respectively sorted with EasySep^®^ human whole blood CD19, CD3, CD66b positive selection kit and human cord blood CD34 positive selection kit following manufacturer’s recommendations.

Fractions were checked for purity by flow cytometry with the MACSQuant^®^ device (Miltenyi Biotec, Germany) using CD20-VioBlue^®^; CD4-(VIT4)-FITC; CD8-PE and CD66abce-APC fluorescent antibodies, following manufacturer’s recommendations. Cell fractions with purity higher than 95% were kept for microchimerism analysis.

### Cord Blood Cell Composition Analyses

CB samples were analyzed for their cell composition with the following combinations of fluorescent antibodies: anti CD20-VioBlue^®^ for B cells, CD3-FITC or PE for T cells, CD4-(VIT4)-FITC and CD8-PE for T helper or cytotoxic T cell subsets respectively, CD66abce-APC for granulocytes, CD16-PE and/or CD56-APC for NK cells, CD45-VioBlue^®^ for all leukocytes and CD34-PE for HPC. Isotype controls were used in parallel. All antibodies were from Miltenyi Biotec, except CD34-PE from STEMCELL™ (STEMCELL™ Technologies, Canada). Analyses were realized on a MACSQuant^®^ device (Miltenyi Biotec, Germany).

### Quantification of Maternal Mc by HLA-Specific Real Time Polymerase Chain Reaction

All DNA samples were evaluated for total DNA concentrations by real-time PCR for β-globin, as previously described (22), using a reference β-globin standard curve common to all the HLA-specific PCR assays. The equivalent DNA of one cell (genome equivalent of 1 cell: 1 gEq) corresponded to 6.6 pg of human DNA. Maternal Mc was quantified in CB DNA samples using primers and probes specific for non-shared, non-inherited HLA-DRB1*01, *15/16, *03, *04, *07, *08, *10, *11, *12, *14 gene sequences, previously validated for specificity and sensitivity (12, 22–24), as well as newly validated HLA-A*01, *02, *11 and DRB1*13 sets, all following the same rigorous validation steps as described previously (23). All primer and probe sets were synthetized by TIB MolBiol (Berlin, Germany). PCR assay sensitivity per reaction-well was of 1 gEq of microchimeric cell in 20,000 gEq of host cells (0.005%), thus DNA samples were adjusted to ~20,000 gEq (132ng) per well and tested for Mc in ten replicate wells (for a combined final sensitivity of 0.0005%). Quantitative PCR assays were done using Light Cycler FastStart DNA MasterPLUS reaction kits on a LightCycler^®^480 instrument (Roche Diagnostics) as previously described (22).

### Statistics

Statistical analyses were conducted using GraphPad Prism 6 software (La Jolla, CA, USA). The non-parametric Mann-Whitney test was used to compare biological, anthropometric or obstetrical parameters (baby’s weight, PAPP-A or βhCG concentrations,…) as well as cord blood cell composition (% of different subsets) between CB positive or negative for maternal Mc. The Chi2 comparison test was used to determine whether type of deliveries, hospital-dependent collection procedures could influence the presence or absence of MMc in CB samples.

To test whether feto-maternal HLA compatibility could influence MMc quantities, CB samples were divided into negative, slightly positive, moderately positive or highly positive categories. The number of maternal cells per million of CB cells found per subset was added up for all the subsets and divided by the number of subsets tested, giving mean values of total MMc per CB ranging from 0.3 to 818 gEq/10^6^. CB were defined as slightly, moderately or highly positive for MMc when mean values of total MMc were respectively, ≤10gEq/10^6^, comprised between 10 and 100 gEq/10^6^ or ≥100gEq/10^6^. When sample sizes were small for comparisons, the Fisher’s exact test was assessed (2X2 or 2x4, http://vassarstats.net/fisher2x4.html). P-values < 0.05 were considered significant.

## Results

### Maternal Mc From Different Cell Types in Cord Blood Samples

**Figure 1:**
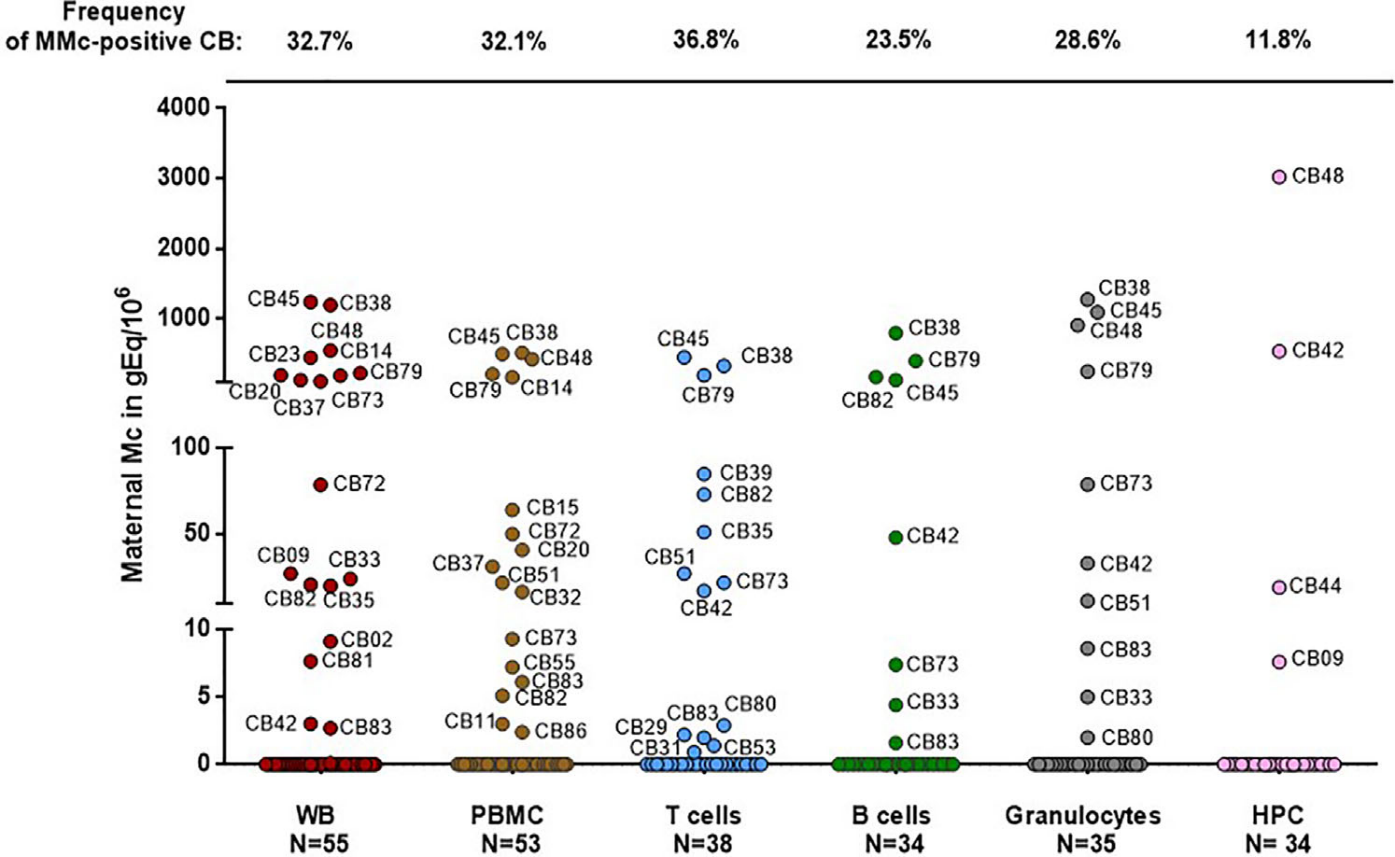
Maternal cells originating from different cell types in cord blood samples. Maternal Microchimerism (MMc), expressed in genome equivalent of cells per million of host cells (gEq/106) is quantified in 55 CB samples. MMc is tested in DNA extracted from whole blood (WB), peripheral blood mononuclear cells (PBMC), T cells (CD3+), B cells (CD19+), granulocytes (CD66+) or from hematopoietic progenitor cells (HPC, CD34+). For example cord blood #45 has 1240 genome equivalent of maternal cells per million (gEq/106) of total cells in whole cord blood and this same cord blood sample has 451 gEq of maternal cells per million of cord blood sorted T cells.

Fifty-five umbilical CB samples from healthy primigravid women have been tested for MMc by NIMA HLA-specific Q-PCR. Mc was assessed in whole blood (WB), and/or PBMC, CD3+ (T cells), CD19+ (B cells), CD66+ (granulocytes) and/or CD34+ (hematopoietic progenitor cells, HPC) fractions (**Figure 1**).

MMc was present in 30 of the 55 samples (55%) in at least one cell compartment/type with the highest frequencies being in sorted T cells (37%), then granulocytes (29%), B cells (24%) and HPC (12%).

Among the 30 samples positive for MMc, four had high quantities of MMc in whole blood comprised between 110 and 305 gEq/10^6^ of cord blood cells (75th percentile of positive values). As expected, the four CB samples having the highest levels in WB had high levels in cell subsets (CB #38, #45, #48 and #79, **Figure 1**) reaching up to 451gEq/10^6^ of sorted T cells, 798 gEq/10^6^ of sorted B cells, 1276 gEq/10^6^ of granulocytes or 3021 gEq/10^6^ of sorted HPC.

### Obstetrical and Anthropometric Parameters That Could Influence Maternal Cell Traffic

As samples were mainly collected in two different hospital maternities, we first tested whether collection procedures could induce differences in MMc detection prevalence between the two centers. There were no significant differences in the prevalence of MMc between CB samples collected in one center or the other, as 14 out of 22 (64%) UCB were positive in the first and 15 out of 32 (47%) in the second (*p=0.22*). Moreover, there was no significant differences in the prevalence of MMc whether the CB was processed within the same day (N=21) or 24 hours after delivery (N=34) with respectively, 67% and 47% (*p=0.16*), nor did the type of delivery influence maternal passage, as two out of the five (40%) CB samples collected after C-section had MMc versus 28 of the 50 samples (56%) collected after vaginal delivery (*p=0.29*). Moreover, quantities of MMc were not higher in positive samples collected after C-section than in positive samples collected after vaginal delivery (data not shown, *p=0.48*). CB samples from children conceived after *in vitro* fertilization treatment did not show differences for MMc compared to CB samples from normal pregnancies.

There was also no effect of the baby’s gender (*p=0.4*), baby’s weight or number of gestation weeks (**Supplementary Figures S1** and **S2**) on the presence or not of maternal cells in CB samples. Of note, but to be taken with caution given the low number, the four CB samples positive for maternal CD34+ cells were from babies heavier than babies whose CB was negative (**Supplementary Figure S3**).

### High Levels of Maternal Circulating Concentrations of Trophoblast-Derived Protein PAPP-A During the First Trimester Correlate With MMc in CB at Delivery

Maternal circulating concentrations of PAPP-A and β-hCG, were measured at 12 (±2) week gestation for 46 of the 55 primigravid healthy women from whom CB was tested.

**Figure 2:**
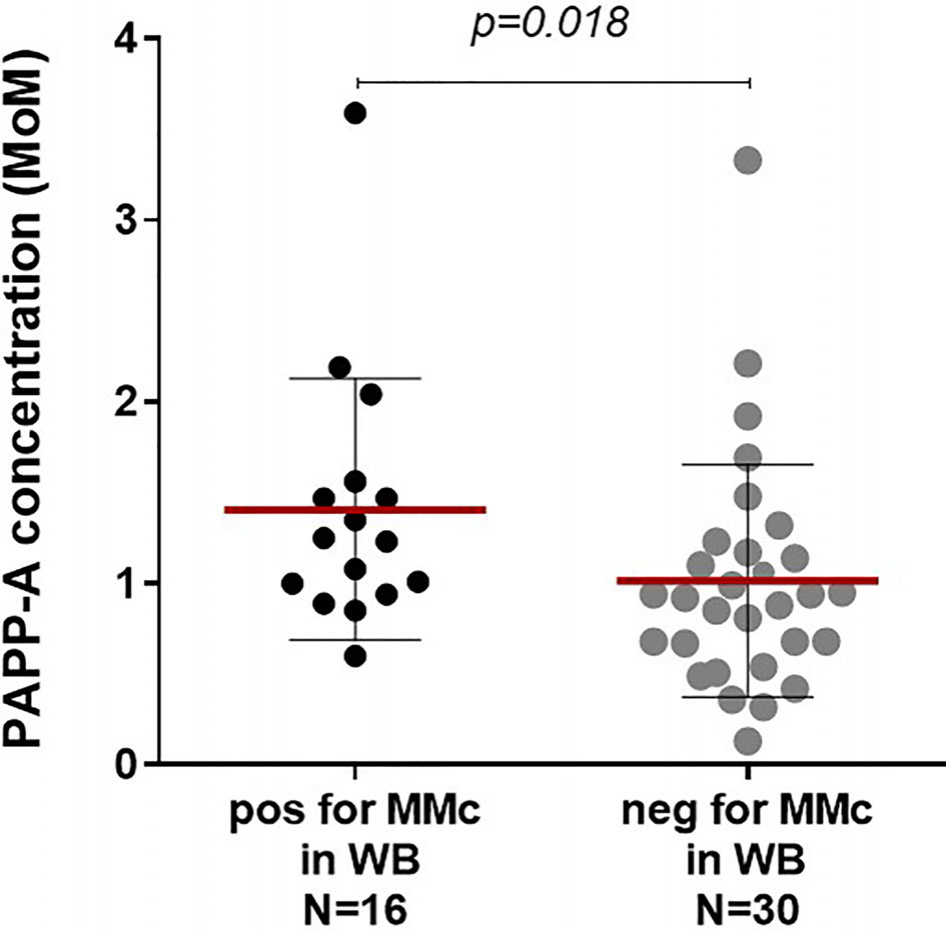
Maternal serological pregnancy-associated protein A (PAPP-A) concentrations are predictive of the presence or not of maternal Mc in CB samples. Cord blood samples are separated into two groups, positive or negative for maternal Mc in whole blood, and both groups analyzed for serological PAPP-A concentrations of the mother at first trimester. PAPP-A concentrations are significantly higher in the positive group than in the negative group (Mann Whitney test, *p=0.018*, with mean concentrations of 1.41 and 1.01 MoM, respectively). Mean concentrations are indicated with red lines and standard deviations with black lines.

High levels of maternal serum PAPP-A concentrations at first trimester significantly correlated with the presence of MMc at delivery in whole cord blood (**Figure 2**, *p=0.018*). As pregnancies conceived using assisted reproductive technologies (ART) have low levels of PAPP-A and could distort the results (25), we did the same analysis excluding four samples issued from ART; results remained significant (*p=0.013*). There was also a significant correlation between maternal PAPP-A quantities and MMc levels in cord blood (Spearman correlation, *p=0.043*, data not shown).

On the other hand, elevated PAPP-A concentrations were not predictive of the presence of MMc in any particular cell subset.

A tendency to lower maternal serum β-hCG concentrations in CB samples positive for MMc was observed but not significantly different from CB negative for MMc (**Supplementary Figure S4**).

### Feto-Maternal HLA-A and/or DR Compatibility Is More Frequent in CB Samples Positive for MMc

**Figure 3:**
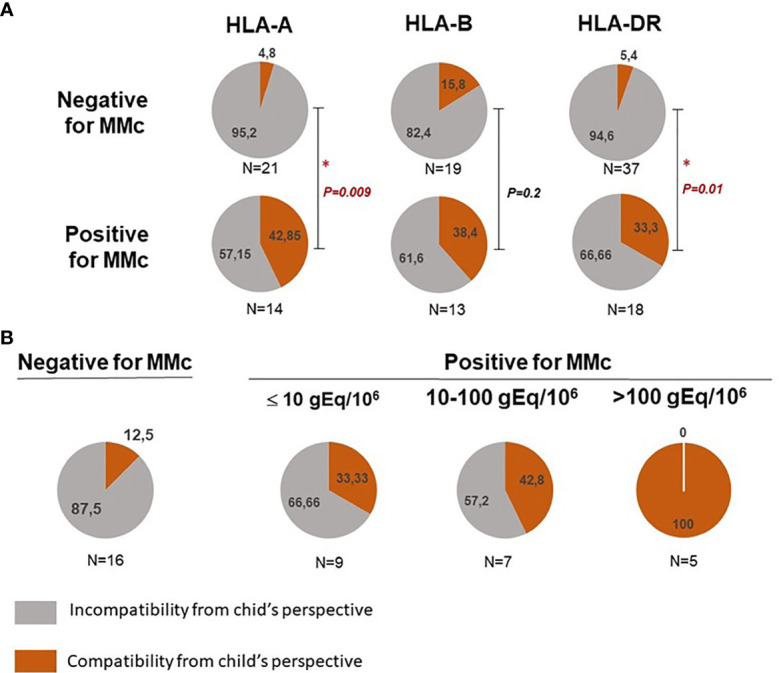
Feto maternal HLA-A, B and/or DR compatibility and presence of maternal Mc in CB samples. (A) Qualitative analyses of HLA-A, B and/or DR compatibility from the child’s perspective with the presence or not of maternal Mc in CB samples. Cord blood samples are separated into negative or positive for MMc in whole blood. The frequency of HLA-A, -B and DR compatible (in red) or incompatible (in grey) CB samples from the child’s perspective is calculated in each group. P values are calculated by comparing compatibility frequencies between negative and positive samples (Two-tailed Fisher’s test 2x2). P values < 0.05 are noted *. (B) CB with the highest quantities of maternal Mc are those for which there is a greater feto-maternal HLA-A and/or DRB1 compatibility from the child’s perspective. Cord blood samples are separated into negative, slightly positive, moderately positive or highly positive for MMc. Slightly positive samples had a mean of MMc per subset tested inferior or equal to 10gEq/106, moderately positive samples had a mean of MMc per subset tested comprised between 10 and 100 gEq/106 and highly positive samples a mean superior to 100gEq/106. The frequency of HLA-A and/or DR compatible (in red) or incompatible (in grey) CB samples from the child’s perspective is calculated in each group. P value is calculated by comparing compatibility frequencies between negative and the three categories positive samples (P = 0.002, Two-tailed Fisher’s test, 2x4).

Mother/child HLA compatibility was classified into either compatibility or incompatibility from the child’s perspective for HLA-A, -B or -DR loci (see **Supplementary Table S2**). CB samples positive for MMc in whole blood were significantly more often HLA-A or DRB1 compatible from the child’s perspective than CB samples negative for MMc (**Figure 3A**, *p=0.009* and *p=0.01* respectively). There was no significant difference for HLA-B compatibility between positive and negative CB.

Moreover, CB with the highest quantities of MMc (See statistical methods) were those for which there was a greater feto-maternal HLA-A and/or DRB1 compatibility from the child’s perspective (**Figure 3B**, *p=0.0072 Fisher’s exact test 2x4*).

### Differences in CB Hematopoietic Cell Composition Between Cord Blood Positive or Negative for MMc

**Figure 4:**
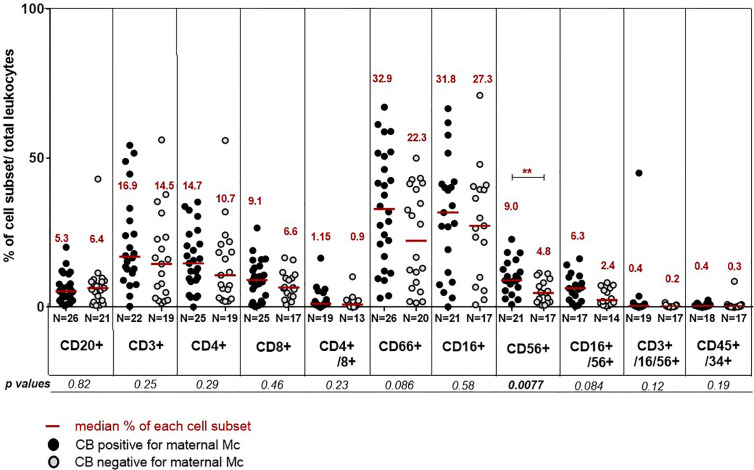
Differences in cord blood hematopoietic cell composition between cord blood positive or negative for MMc. Cord blood are separated into positive for MMc (black circles) or negative for MMc (grey circles) and their respective percentage of different cell types evaluated. The following combinations of fluorescent antibodies are used to define the different cell populations: anti CD20-VioBlue^®^ for B cells, CD3-FITC for T cells, CD4-(VIT4)-FITC and CD8-PE for T helper or cytotoxic T cell subsets respectively, CD66abce-APC for granulocytes, CD16-PE and/or CD56-APC for NK cells, CD3-FITC/CD16-PE/CD56-APC for NKT cells, CD45-VioBlue^®^ for all leukocytes and CD34-PE for hematopoietic progenitor cells. P values <0.01 are noted **.

Finally, we asked whether the presence of MMc in cord blood samples could have an influence on their hematopoietic cell composition. Cord blood units positive for MMc consistently trended toward containing more T cells (CD3+ in general, or specifically CD4+ and CD8+ cells), granulocytes (CD66+), monocytes (CD16+), NK cells (CD56+ or CD16+/CD56+), but were only significantly enriched in CD56+ cells compared to CB units negative for MMc (**Figure 4**). An inversed tendency, although not significant, was observed for B cells, being enriched in CB negative for MMc.

## Discussion

Since the first successful umbilical cord blood transplantation in 1989 to treat a child suffering from severe Fanconi anemia (26), allogeneic hematopoietic stem cells from CB samples have increasingly been used as a curative treatment for many cancers and inherited non-malignant diseases (27). CB transplantation requires less stringent donor/recipient HLA matching than other sources of hematopoietic stem cells, which is a strong advantage, knowing that almost three quarters of patients do not have an HLA-matched alternative source (28). From a practical point of view, CB samples have also the advantage to be easily cryopreserved and banked. Because the most serious issue in cord blood banking is the cost, it remains to be seen on what criteria some should be stored rather than others (29).

Over sixty years ago, several authors had demonstrated, by tagging, prior to delivery, maternal erythrocytes with radioactive elements, that maternal cells could cross the placenta and be found in infant’s peripheral blood or in CB units (30). Since then, the presence of nucleated maternal cells in CB samples has been studied with non-invasive methods, thanks to PCR assays among others, and MMc is obviously a common phenomenon in CB samples (7, 10, 12–14, 31–34).

The current study has the particularity to test MMc from primigravid women. We showed that 33% of CB contained MMc in whole blood and the frequency was up to 55% when looking for its presence in whole blood and/or at least one of the T, B, granulocyte or hematopoietic progenitor cell subsets. Compared to the most recent studies using real time PCR assays (12, 13), our results are slightly lower than those obtained by Kanaan et al. (12) (52.9% in whole blood and 85.2% in any cell subset), but higher than those obtained from Kanold et al. (13) who showed only 11% of CB being positive for MMc even when testing cell subsets. Differences are likely coming from sensitivity of the Q-PCR assays. Kanaan et al. used the same highly sensitive panel of HLA-specific QPCR assays we previously developed to target NIMA (23), while Kanold et al. used sequence polymorphism systems having less sensitivity (0.01%) (13). Another very likely influence, could be gravidity of women from whom CB were tested. Although the pregnancy history was not always provided in previous reports, to our knowledge, this is the only study testing CB *only* from primigravid women. This arbitrary choice was i) to avoid any other confounding source of Mc (i.e. from an older brother or sister passing through maternal flow), ii) because primigravidity is a recommended criteria for CB transplantation as CB from primigravid women are associated with larger blood volume and higher cellular content, pledge of transplantation success (35).

Importantly and contrary to what was initially thought, CB samples carrying maternal Mc are likely to be a better option for transplantation, as graft-versus-leukemia effect may be mediated by maternal cells (16). For 37% of CB samples, MMc was found in T lymphocytes, in agreement with recently described greater MMc quantities in memory T cells (12). Nevertheless, MMc was not quantitatively higher in any particular cell subset and was found across all subsets tested. MMc was found among sorted HPC (CD34+) in 4/34 CB, with levels reaching up to 0.3% of maternal CD34+ cells in one CB. Giving that adequate CD34+ cell doses in CB transplantation for hematologic malignancies has been evaluated at ≥1,5 x10^5^/kg (2), using such maternally enriched CD34+ cell CB as the one described here, would mean that a 70kg adult recipient would receive a non-negligible amount of 3,000 maternal CD34+ cells. As expected, CB samples being highly positive for MMc in whole blood were systematically positive in cell subsets, which may provide a practical advantage for rapid screening of MMc in CB.

Two biological and immunological parameters were significantly correlated with the presence and quantity of MMc: maternal serum PAPP-A concentration at first trimester and feto-maternal HLA-A and DRB1 compatibility.

PAPP-A (or papalysin 1) is a secreted metalloproteinase produced by the fetal syncytiotrophoblast cells and subsequently released in the maternal circulation. PAPP-A activates the insulin-like growth factor (IGF) pathway (36) and is an important protein in promoting decidual vascularization (37). Decreased first trimester PAPP‐A concentrations are a predictor of adverse pregnancy outcome, with a significant reduction of the placental volume and its vascularization (38). Conversely, high levels of PAPP-A have generally no pregnancy consequences, except cases of placenta accretes (39). We showed that high levels of maternal serum PAPP-A concentrations at first trimester were significantly predictive of the presence of MMc at delivery in whole cord blood. Such correlation, never reported before, seems to indicate that the pressure exerted on the maternal vascular system by fetal trophoblast cells secreting large amounts of PAPP-A promotes feto-maternal exchange resulting in higher quantities of MMc in CB and possibly in the child’s circulation.

Maternal-fetal histocompatibility has been one of the first proposed factor to regulate the presence and quantities of naturally occurring maternal cells in the progeny. It comes from observations in the field of transplantation, where the HLA relationship between donor and recipient is of importance as chronic GvHD is more likely when the donor is homozygous for an HLA allele for which the recipient is heterozygous, giving compatibility from the recipient’s perspective. Indeed, when testing MMc in whole blood samples from 30 informative second- and third-trimester fetuses, Berry et al. showed that MMc in fetal blood was associated with maternal HLA-DRB1 and/or DQB1 compatibility from child’s perspective (40). However, no such study has been done in cord blood because mothers are rarely HLA typed. Similarly to what was found in fetuses, our results showed that high levels of maternal Mc in cord blood were associated with maternal compatibility from the child’s perspective at the HLA-DRB1 loci, but also the HLA-A loci.

Compatibility from the child’s perspective leads to a capacity of maternal (donor) lymphocytes to evade HLA-mediated immune detection by the child (host), but a preserved ability of maternal (donor) lymphocytes to recognize and react to host tissues. Interestingly, child-to-mother HLA-A compatibility has been previously found in children with Biliary Atresia (BA), an inflammatory cholangiopathy, for which high levels of MMc have been identified in the livers of patients (41, 42). This suggests that the particular HLA relationship of patients with BA with their mothers may result in an increase in MMc. It remains to be seen whether such an HLA relationship dangerously increases the maternal-fetal exchange to the point of later triggering an autoimmune disease in the recipient. Yet, two recent studies investigated whether MMc measured in cord blood could predict the risk of future celiac disease or type 1 diabetes in children but did not find an association (43, 44).

Finally and importantly, we asked whether the presence of MMc in cord blood samples could have an influence on the hematopoietic composition of fetal cells. We found that CB samples positive for MMc had an enriched composition of immune fetal cells, in particular CD56+ cells compared to CB negative.

CD56 is the phenotypic marker of natural killer cells (NK cells). Interestingly, CB units with higher doses of nucleated cells have been associated with faster engraftment and better overall survival (2, 29). Moreover, NK cells are known to be the main effector for GVL reactions early after hematopoietic stem cell transplantation and are enriched in umbilical cord blood compared to peripheral blood (45). It is to note that if a CB sample carrying maternal Mc is enriched in CD56 positive cells it does necessary mean that the CD56+ cell subset is enriched in maternal cells. On the contrary, the increased number of CD56+ cells in CB positive for MMc is unlikely coming from MMc itself because of their small number, but rather, MMc could influence the number of CD56+ fetal cells. Supporting this hypothesis, in a previous study from Kanaan et al., maternal cells were not increased in the NK subset (12).

Although CD56 is the surface antigen that characterizes NK cells, it can also be expressed by many more immune cells, including alpha beta T cells, gamma delta T cells, dendritic cells, and monocytes (46). Thus, it would be interesting to further analyze, with other surface markers and gating strategies, which particular cell fraction is increased among the CD56 positive cells in CB positive for MMc.

Another important subset to analyze, in a context of maternal cells, would be the regulatory T cells. Indeed, Mold et al. reported that the human fetal immune system generates regulatory T cells (CD41CD25highFoxP3Tregs) that suppress fetal immune responses to maternal antigens, and that this tolerance persists at least until early adulthood (47).

In conclusion, we have identified factors predicting the presence of maternal cells in CB samples; we have a better understanding of the immune composition of CB samples in the context of maternal cells. These findings are important issues for CB banking strategies. Indeed, clinical practice may be improved by selecting CB from women with elevated PAPP-A concentrations and/or issued from HLA-compatible pairs, to increase the chances of obtaining a MMc-enriched CB, pledge of transplantation success.

## Data Availability Statement

The raw data supporting the conclusions of this article will be made available by the authors, without undue reservation.

## Ethics Statement

The studies involving human participants were reviewed and approved by CPP Sud-Méditerranée II. The patients/participants provided their written informed consent to participate in this study.

## Author Contributions 

MH, KK, and NCL conceived and designed the experiments. MH, KK, MM, GM, and NCL performed the experiments. NCL analyzed the data. X-CD, FB, NL, J-FC, and RD contributed to recruitment of healthy primigravid women and their mother. X-CD, CP, and J-FC contributed to clinical, anthropological, and/or obstetrical data. NCL wrote the paper. JR reviewed the manuscript and made corrections. All authors contributed to the article and approved the submitted version.

## Funding

This study was supported for equipment and post-doctoral salaries by the Provence-Alpes-Côte d’Azur (PACA) region as part of the open call for exploratory projects (APEX grant # 2012_06549E, 2012_11786F and 2014_03978) and the Foundation for Medical Research (FRM) as part of the “Hope for Research” program, “Aid for innovative projects: Funding for an Engineer” (Grant #ING20140129045).

## Conflict of Interest

The authors declare that the research was construed in the absence of any commercial or financial relationships that could be constructed as a potential conflict of interest.
