# Corrigendum: Factors Predicting the Presence of Maternal Cells in Cord Blood and Associated Changes in Immune Cell Composition

**DOI:** 10.3389/fimmu.2021.763236

**Published:** 2021-10-01

**Authors:** Marina El Haddad, Karlin Karlmark, Xavier-Côme Donato, Gabriel Martin, Florence Bretelle, Nathalie Lesavre, Jean-François Cocallemen, Marielle Martin, Christophe Picard, Jean Roudier, Raoul Desbriere, Nathalie C. Lambert

**Affiliations:** ^1^ INSERM UMRs 1097 Arthrites Autoimmunes, Aix Marseille Université, Marseille, France; ^2^ Department of Obstetrics and Gynecology, St Joseph Hospital, Marseille, France; ^3^ Department of Gynaecology and Obstetrics, Pôle Femme Enfant, AP-HM, Assistance Publique-Hôpitaux de Marseille, AMU, Aix-Marseille Université, France; ^4^ CIC1409, AMU, AP-HM, Marseille, France; ^5^ Centre National de la Recherche Scientifique (CNRS) UMR7268 (ADES), “Biologie des Groupes Sanguin”, Marseille, France; ^6^ Etablissement Français du Sang (EFS), Marseille, France; ^7^ Service de Rhumatologie, Hôpital Sainte Marguerite, AP-HM, Marseille, France

**Keywords:** cord blood, maternal microchimerism, PAPP-A, HLA compatibility, NK cells, transplantation

In the original article, there was a mistake in the legend for **Figure 1** as published. The value of MMc in WB for CB#45 indicated in the legend of **Figure 1** corresponded to the value of MMc (305 gEq) per number of host cells tested per experiment (~246, 000 gEq), not per million of host cells. The correct legend appears below.

**Figure 1:**
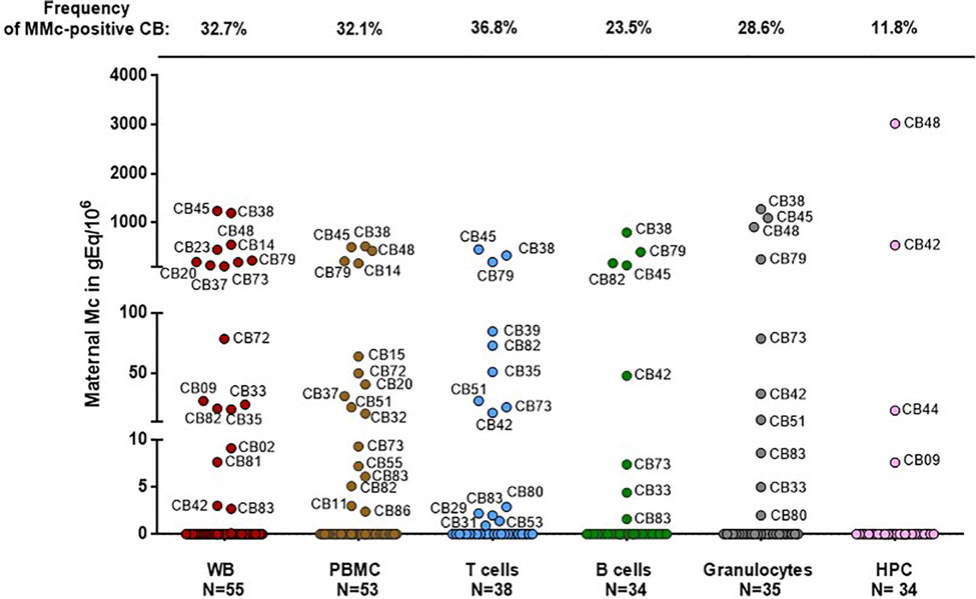
Maternal cells originating from different cell types in cord blood samples. Maternal Microchimerism (MMc), expressed in genome equivalent of cells per million of host cells (gEq/106) is quantified in 55 CB samples. MMc is tested in DNA extracted from whole blood (WB), peripheral blood mononuclear cells (PBMC), T cells (CD3+), B cells (CD19+), granulocytes (CD66+) or from hematopoietic progenitor cells (HPC, CD34+). For example cord blood #45 has 1240 genome equivalent of maternal cells per million (gEq/106) of total cells in whole cord blood and this same cord blood sample has 451 gEq of maternal cells per million of cord blood sorted T cells.

The correct legend appears below **Figure 1**.

In the original article, there was a mistake in the legend for **Figure 3B** as published. As the values of MMc in WB indicated corresponded to the values of MMc per number of host cells tested per experiment (mean ~212,360 gEq ±31,110), not per million of host cells, this results in minimal changes in the p value. Moreover the symbols “inferior or equal to” or “superior to” did not appear correctly, they are spelled out in the corrected legend. The correct legend appears below.

**Figure 3:**
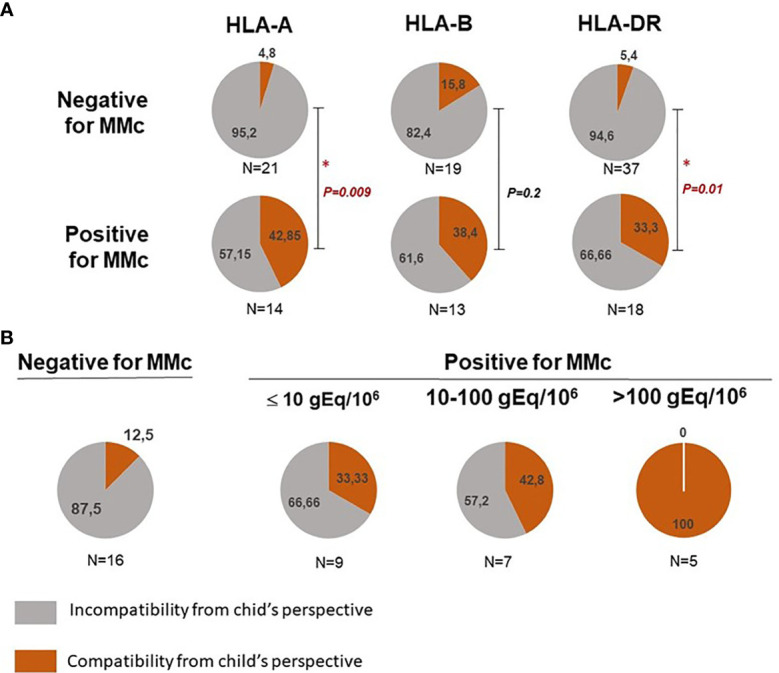
Feto maternal HLA-A, B and/or DR compatibility and presence of maternal Mc in CB samples. **(A)** Qualitative analyses of HLA-A, B and/or DR compatibility from the child’s perspective with the presence or not of maternal Mc in CB samples. Cord blood samples are separated into negative or positive for MMc in whole blood. The frequency of HLA-A, -B and DR compatible (in red) or incompatible (in grey) CB samples from the child’s perspective is calculated in each group. P values are calculated by comparing compatibility frequencies between negative and positive samples (Two-tailed Fisher’s test 2x2). P values <0.05 are noted *. **(B)** CB with the highest quantities of maternal Mc are those for which there is a greater feto-maternal HLA-A and/or DRB1 compatibility from the child’s perspective. Cord blood samples are separated into negative, slightly positive, moderately positive or highly positive for MMc. Slightly positive samples had a mean of MMc per subset tested inferior or equal to 10gEq/106, moderately positive samples had a mean of MMc per subset tested comprised between 10 and 100 gEq/106 and highly positive samples a mean superior to 100gEq/106. The frequency of HLA-A and/or DR compatible (in red) or incompatible (in grey) CB samples from the child’s perspective is calculated in each group. P value is calculated by comparing compatibility frequencies between negative and the three categories positive samples (P= 0.002, Two-tailed Fisher’s test, 2x4).

The correct legend appears below **Figure 3**.

In the original article, there was a mistake in **Figure 1** as published. As explained above, the values of MMc in WB indicated in the original article corresponded to the values of MMc per number of host cells tested per experiment (mean ~212,360 gEq ±31,110), not per million of host cells. The mistake was only for values in whole blood. All the other values in T cells, B cells, granulocytes and CD34+ cells are correct. The corrected **Figure 1** appears below.

In the original article, there was a mistake in **Figure 3B** as published. Consequent to the initial mistake where values of MMc in WB were given per number of host cells tested per experiment instead of MMc per million of host cells, instead of having 11 cord blood (CB) samples with MMc ≤10 gEq, six between 10-100 gEq and four with > 100 gEq, respectively N=9, N=7 and N=5 CB corresponded to each category. Results are extremely similar to previously published data and similarly significant (P=0.002). The corrected **Figure 3B** appears below.

In the original article, there was an error in **Patients and Methods, Statistics**, paragraph 2 as published: “The number of maternal cells per million of CB cells found per subset was added up for all the subsets and divided by the number of subsets tested, giving mean values of total MMc per CB ranging from 0.3 to 744 gEq/10^6^”. The values of MMc in WB indicated in the text corresponded to the values of MMc per number of host cells tested per experiment (mean ~212,360 gEq ±31,110), not per million of host cells.

A correction has been made to the text as follows: “The number of maternal cells per million of CB cells found per subset was added up for all the subsets and divided by the number of subsets tested, giving mean values of total MMc per CB ranging from 0.3 to 818 gEq/10^6^.”

In the original article, there was an error in **Results**, paragraph 3 as published: “Among the 30 samples positive for MMc, four had high quantities of MMc in whole blood comprised between 110 and 305 gEq/10^6^ of cord blood cells (75th percentile of positive values)”.

A correction has been made to the text as follows: “Among the 30 samples positive for MMc, four had high quantities of MMc in whole blood comprised between 226 and 1240 gEq/10^6^ of cord blood cells.”

The authors apologize for these errors and state that this does not change the scientific conclusions of the article in any way. The original article has been updated.

## Publisher’s Note

All claims expressed in this article are solely those of the authors and do not necessarily represent those of their affiliated organizations, or those of the publisher, the editors and the reviewers. Any product that may be evaluated in this article, or claim that may be made by its manufacturer, is not guaranteed or endorsed by the publisher.

