# Supplementary material for: Factors Predicting the Presence of Maternal Cells in Cord Blood and Associated Changes in Immune Cell Composition
Source: Front Immunol. 2021 Apr 22;12:651399. doi: 10.3389/fimmu.2021.651399 (PMC8100674; doi:10.3389/fimmu.2021.651399)
Supplement: Supplementary file 1 [file Image_1.pdf]

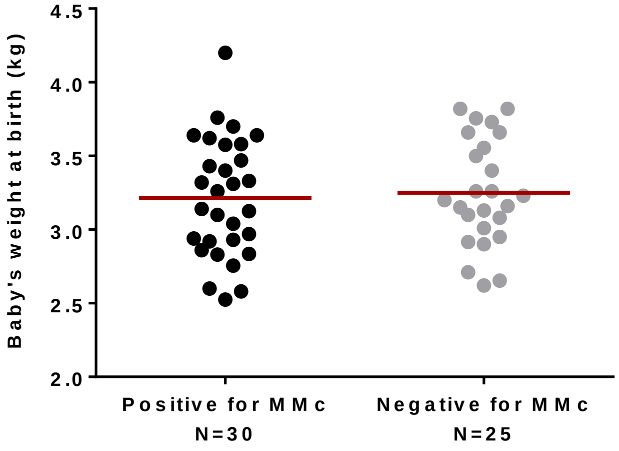


**Supplementary Figure S1. Baby's weights at delivery in cord blood samples positive or negative for maternal microchimerism (MMc).** Cord blood samples are separated into two groups, positive or negative for MMc in any cell subset tested, and both groups analyzed for the weights of the babies. Mean weights of babies are indicated with red lines in the positive and the negative group (Mann Whitney test, two-tailed *p=0.62*).
