# Supplementary material for: Factors Predicting the Presence of Maternal Cells in Cord Blood and Associated Changes in Immune Cell Composition
Source: Front Immunol. 2021 Apr 22;12:651399. doi: 10.3389/fimmu.2021.651399 (PMC8100674; doi:10.3389/fimmu.2021.651399)
Supplement: Supplementary file 2 [file Image_2.pdf]

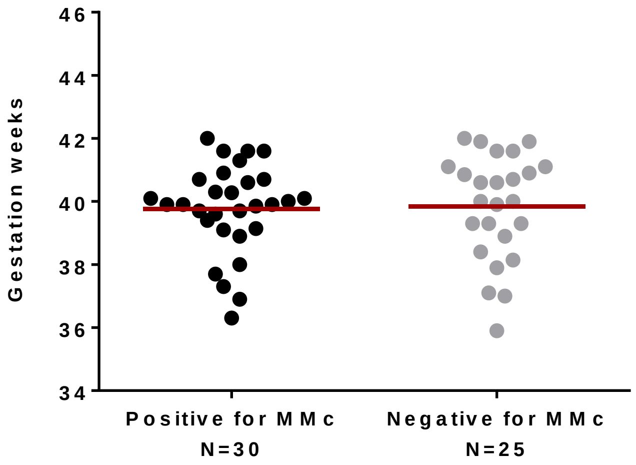

Supplementary Figure S2. Number of gestation weeks in cord blood samples positive or negative for maternal microchimerism (MMc). Cord blood samples are separated into two groups, positive or negative for MMc in any cell subset tested, and both groups analyzed for gestation weeks of the mother. Mean numbers of gestation weeks are indicated with red lines in the positive and the negative group (Mann Whitney test, two-tailed *p=0.65*).
