# Supplementary material for: Factors Predicting the Presence of Maternal Cells in Cord Blood and Associated Changes in Immune Cell Composition
Source: Front Immunol. 2021 Apr 22;12:651399. doi: 10.3389/fimmu.2021.651399 (PMC8100674; doi:10.3389/fimmu.2021.651399)
Supplement: Supplementary file 3 [file Image_3.pdf]

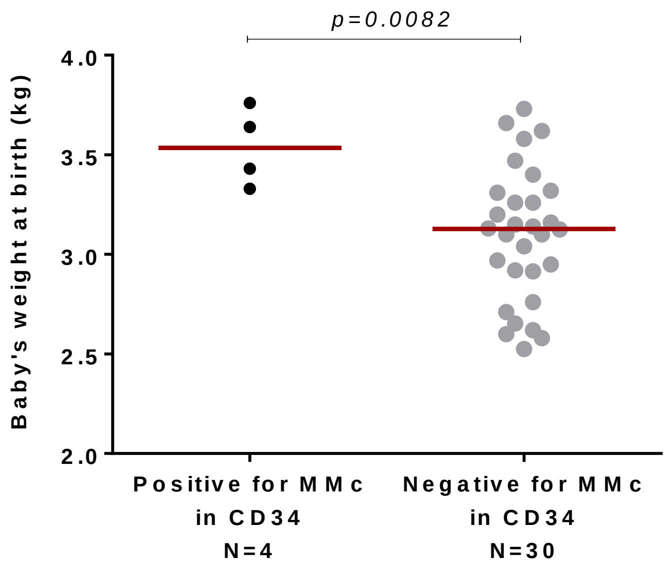

Supplementary Figure S3. Baby's weights at delivery in cord blood samples positive or negative for maternal microchimerism (MMc) in CD34+ cell subset. Cord blood samples are separated into two groups, positive or negative for MMc in CD34+ cell subset (hematopoietic progenitor cells), and both groups analyzed for the weights of the babies. Mean weights of babies are indicated with red lines in the positive and the negative group and are respectively 3.540 and 3.090 kg (Mann Whitney test, two-tailed *p=0.0082*).
