# Supplementary material for: Factors Predicting the Presence of Maternal Cells in Cord Blood and Associated Changes in Immune Cell Composition
Source: Front Immunol. 2021 Apr 22;12:651399. doi: 10.3389/fimmu.2021.651399 (PMC8100674; doi:10.3389/fimmu.2021.651399)
Supplement: Supplementary file 4 [file Image_4.pdf]

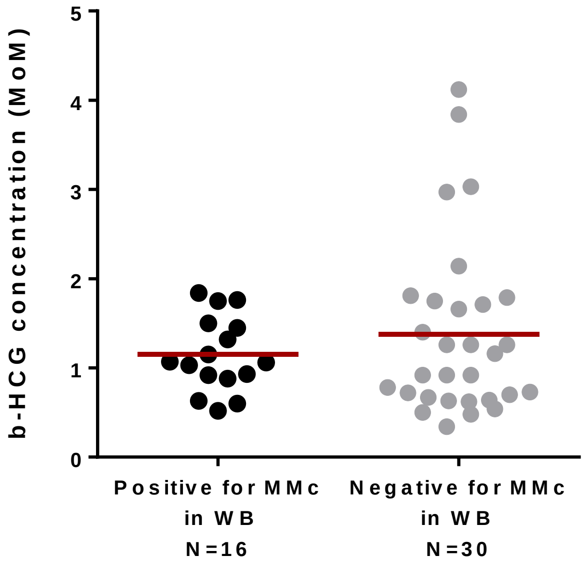

Supplementary Figure S4. Maternal serological free beta human chorionic gonadotropin (β-hCG) concentrations in cord blood samples positive or negative for maternal microchimerism (MMc). Cord blood samples are separated into two groups, positive or negative for MMc in whole blood, and both groups analyzed for serological β-hCG concentrations of the mother at first trimester. Mean concentrations are indicated with red lines and are respectively in the positive and the negative group 1.15 and 1.37 MoM (Mann Whitney test, two-tailed *p=0.9*).
