# Supplementary material for: Factors Predicting the Presence of Maternal Cells in Cord Blood and Associated Changes in Immune Cell Composition
Source: Front Immunol. 2021 Apr 22;12:651399. doi: 10.3389/fimmu.2021.651399 (PMC8100674; doi:10.3389/fimmu.2021.651399)
Supplement: Supplementary file 5 [file Table_1.pdf]

Obstetrical, clinical parameters	Details
History of pregnancies N=55	55 pregnancies for which no fetal abnormalities were observed and a single live fetus was delivered<br>40 normal pregnancies*<br>1 membrane rupture at 34 weeks of amenorrhea<br>1 hypothyroidism/ IVF (sperm donation)<br>1 IVF (sperm donation)<br>1 IVF (gamete donation: unknown)<br>1 IVF (no gamete donation)<br>1 endometriosis/ IVF (ova donation and intracytoplasmic sperm injection) / mild high blood pressure<br>1 polycystic ovary syndrome
History of deliveries N=55	5 delivered a baby with birth weight above the 10th centile of normal for gestational age<br>3 delivered before 37 complete weeks of gestation<br>55 single lived fetus delivered
PAPP-A (MoM) (N=46)	1.18 (range: 0.13 - 3.9)
βhCG (MoM) (N=46)	1.31 (0.34 - 4.12)
Type of deliveries	50 vaginal, 5 caesarian
Number of girls<br>Number of boys	21<br>34
Mean weight of baby males (kg)<br>Mean weight of baby females (kg)<br>Mean weight of all babies (kg)	3.25 (range 2.53- 4.20)<br>3.20 (range 2.62- 3.76)<br>3.23 (range 2.53 - 4.20)
Mean number of gestational weeks	39.8 (range: 35.9 - 42.0)
Mean maternal age	29.5 (range: 20.6 - 40.5)

* A normal pregnancy was defined as a pregnancy in which a single live fetus was delivered after 37 complete weeks of gestation with birth weight above the 10th centile of normal for gestational age and for which no fetal anomalies were observed.
IVF: in vitro fecundation; PAPP-A: Pregnancy Associated Plasma Protein A; bhCG: beta human chorionic gonadotropin; MoM : multiple of the median.

Supplementary Table S1- obstetrical, anthropometric and clinical characteristics of the 55 primigravid mothers and children from whom cord blood samples were collected
