# Supplementary material for: Factors Predicting the Presence of Maternal Cells in Cord Blood and Associated Changes in Immune Cell Composition
Source: Front Immunol. 2021 Apr 22;12:651399. doi: 10.3389/fimmu.2021.651399 (PMC8100674; doi:10.3389/fimmu.2021.651399)
Supplement: Supplementary file 6 [file Table_2.pdf]

**Examples for HLA-DRB1**	**Compatibility from child's perspective**		**Incompatibility from child's perspective**	
	Identical DRB1 genotypes	Homozygous mother's DRB1	Different DRB1 genotypes	Homozygous child's DRB1
**mother**	***01/*15**	***01/*01**	***01/*03**	***01/*15**
**child**	***01/*15**	***01/*15**	***01/*15**	***01/*01**

Supplementary Table S2. Feto-maternal HLA compatibility. In the current table HLA-DRB1 compatibility is used as an example. There are only four possibilities of HLA compatibility between a mother and her child. In the two first cases, the *compatibility from the child's perspective* is either due HLA-DRB1 genotype sharing between mother and child or due to mother's HLA-DRB1 homozygosity. In both cases the child will not recognize any HLA-DRB1 from the mother as foreign. Inversely, in the two last cases, the *incompatibility from the child's perspective* is either due to different HLA-DRB1 genotypes between the mother and the child or to child's HLA-DRB1 homozygosity. In both cases the child will recognize as foreign HLA-DRB1*03 or *15 from the mother.
